# Long-Term Followup Comparing Two Treatment Dosing Strategies of ^**125**^I Plaque Radiotherapy in the Management of Small/Medium Posterior Uveal Melanoma

**DOI:** 10.1155/2013/517032

**Published:** 2013-02-28

**Authors:** Timothy G. Murray, Arnold M. Markoe, Aaron S. Gold, Fiona Ehlies, Ernesto Bermudez, Andrea Wildner, Azeema Latiff

**Affiliations:** ^1^Murray Ocular Oncology and Retina Practice, 6705 Red Road, Suite 412, Miami, FL 33143, USA; ^2^Department of Radiation Oncology, University of Miami School of Medicine, Miami, FL, USA

## Abstract

*Objective*. To investigate the efficacy of two different dosing strategies of radioactive iodine-125 (^125^I) in the management of small- and medium-sized posterior uveal melanoma. *Patients and Methods*. The medical records of consecutive patients with choroidal melanomas between 1.5 and 5.0 mm in apical height treated initially with ^125^I plaque radiotherapy were reviewed. Patients were treated with one of the following two treatment dosing strategies: (1) 85 Gy to the apical height of the tumor (group 1) or (2) 85 Gy to a prescription point of 5.0 mm (group 2). *Results*. Of 95 patients, 55 patients were treated to the apical height of the tumor, and 40 were treated to a prescription point of 5.0 mm. Comparative analysis of the incidence rates of specific complications between the two groups demonstrates that group 2 had a significantly higher incidence of radiation retinopathy, radiation optic neuropathy, and/or visually significant cataract formation than group 1 (*P* = 0.028). *Conclusion*. Treatment of choroidal melanomas less than 5 mm in apical height with ^125^I brachytherapy to the true apical height is equally effective when compared to treatment with 85 Gy to 5.0 mm. Treatment to the apical height of the tumor may result in lower incidence of radiation-related complications.

## 1. Introduction

Choroidal melanoma is the most common primary malignant intraocular tumor in adults, with an estimated incidence in the United States of 6 cases per million persons [1, 2]. Before the advent of plaque radiotherapy, enucleation was the standard treatment for these tumors. More recently, radioactive iodine 125 (^125^I) brachytherapy has gained acceptance as an effective treatment alternative for small- and medium-sized melanomas [3–7]. This globe-preserving treatment has been shown to be equally effective as enucleation in local tumor control and prevention of metastasis while often sustaining useful vision among patients with medium-sized choroidal melanomas [8, 9]. However, patients may experience sight-threatening complications of plaque irradiation including radiation retinopathy, radiation papillopathy, cataract, and neovascular glaucoma [3, 10–13]. Radiation-related vascular occlusions have been shown to be dose-dependent [14, 15]. It may be postulated, and therefore, that using lower doses of radiation would lower the incidence of treatment-related complications [11]. The optimal radiation dose for choroidal melanoma remains unknown. Conventional treatment is based largely upon dosage regimens used in the Collaborative Ocular Melanoma Study (COMS), in which tumors were treated with 85 Gy to a minimum of 5.0 mm from the inner sclera for all tumors less than 5.0 mm in apical height [16–18]. The purpose of the current study is to investigate the efficacy, complication rates, and visual outcomes of treating small and medium choroidal melanomas with ^125^I plaque radiotherapy with a prescription dose to the actual apical tumor height. These results are compared with those from patients treated with standard COMS dosing.

## 2. Patients and Methods

The study protocol received Institutional Review Board (IRB) approval for a retrospective clinical study involving human subjects. The records of all patients with choroidal melanomas between 1.5 and 5.0 mm in apical height who were examined and treated at the Oncology Service of the Bascom Palmer Eye Institute between February 1, 1991 and April 1, 1998, were reviewed. All patients received ^125^I plaque radiotherapy as primary treatment using one of the two dosing strategies. Patients ineligible for the COMS study with tumor heights <2.5 mm or refusing COMS radiation dosing regiments were treated to true apical height (group 1). Patients participating in the COMS study were treated to a prescription point of 5.0 mm (group 2). All patient data was reevaluated at a ten-year followup window. 

All patients were evaluated for metastatic disease prior to treatment and upon followup. Metastatic workup included physical examination, liver enzymes, abdominal imaging, and chest X-ray. Patients with abnormal liver function tests underwent abdominal imaging, including ultrasound, MRI, and/or CT. Patients were examined with indirect ophthalmoscopy, as well as A- and B-scan ultrasonography.

Episcleral plaques were applied using standard surgical techniques described previously, and intraoperative echographic localization of the plaque was performed in all cases [19–21]. 

The following clinical variables were recorded for each patient at the time of initial examination: age, gender, involved eye, medical and ocular history, previous ocular surgeries, visual acuity, intraocular pressure, tumor characteristics, and associated clinical findings. The specific tumor characteristics recorded included tumor shape, location, basal dimension as determined clinically, tumor apical height in mm (measured on B-scan ultrasonography), retinal pigment epithelial changes or drusen overlying the tumor, orange pigmentation, tumor pigmentation, and presence of orbital involvement. Information about the development of local tumor recurrence (defined as clinically or echographically documented growth requiring further treatment), evidence of metastasis, occurrence of treatment-related complications, and any additional treatment administered (i.e., enucleation) was recorded from each followup visit at 1, 3, 6, 12, 24, and 48 months. Furthermore, patients from both groups that presented with sight threatening complications during followup, such as macular edema, were treated with anti-VEGF or intravitreal steroidal therapy.

## 3. Results

The study included 54 females (53.5%) and 47 males (46.5%); 88 (87.1%) patients were Caucasian, 12 (11.9%) were Hispanic, and 1 was non-Caucasion, non-Hispanic. Of the 95 tumors, 54 (53.5%) involved the right eye, and 47 (46.5%) involved the left eye. When patient demographics, systemic diseases, and preexisting ocular pathology were compared between the two treatment groups, the incidence of age-related macular degeneration was significantly higher in patients from group 1 than group 2, (16% and 2.5%, resp., *P* = .03).

 Of the 95 patients identified, 55 patients received treatment to the actual apical height of the tumor (group 1), and 40 patients received treatment to a prescription point of 5.0 mm (group 2). Pearson's chi-squared test was used for comparative analysis. The mean followup interval was 149.7 months overall, 145.2 months for group 1 compared with 151.1 months for group 2 (*P* = 0.9). The tumor size averaged 10.7 mm in diameter and 3.3 mm in height for group 1 and 10.8 mm in diameter and 3.2 mm in height for group 2 (Table 1). Tumor shape and location for each treatment group are summarized in Table 1.

**Figure 1:**
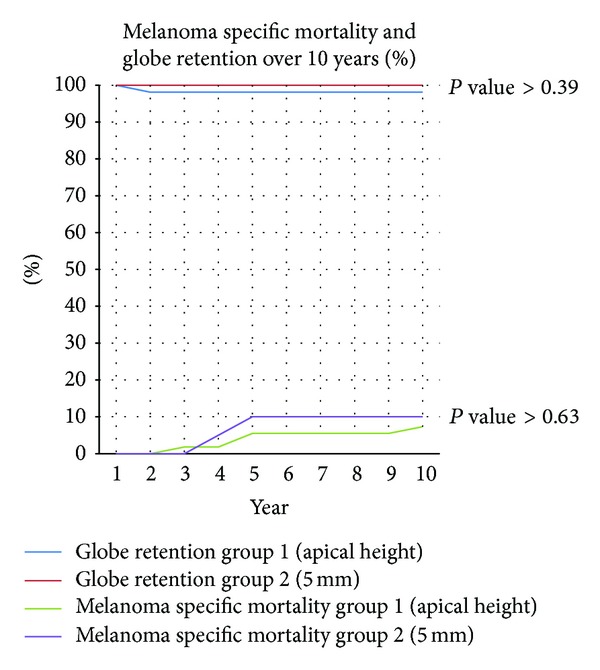


Local diffuse tumor recurrence developed in one patient from group 1, occurring ten months after treatment. There were no documented recurrences during the followup interval in group 2. This difference was not significant (*P* = 0.4). Only eight patients experienced metastasis during the followup period, four from each treatment group (Figure 1). All eight patients died during the followup period.  Table 2 summarizes these findings.

There was no significant difference in visual acuity outcomes at 6, 12, and 24 months between the two treatment groups (*P* = 0.5, 0.7, and 0.5, resp.) (Table 3). There was also no significant difference between groups in the percentage of patients with worsening vision (loss of 2 or more lines on the Snellen acuity chart) at 6, 12, and 24 months (*P* = 0.6, 0.5, and 0.4, resp.).

Fifty-three patients developed at least one of the following radiation complications: radiation retinopathy (45), radiation papillopathy (23), cataract (13), and vitreous hemorrhage (7); 14 (27%) patients that experienced at least one of these complications were from group 1; 39 (98%) were from group 2 (*P* < 0.001) (Table 4). Other treatment-related complications experienced were strabismus (14 patients) and exudative retinal detachment (3 patients) (Table 4). The incidence of each complication was compared between treatment groups. The incidence of radiation retinopathy and cataract was significantly higher in group 2 when compared with group 1; the incidence of other complications was not significantly different between treatment groups (Table 4). 

## 4. Comment

Clinical studies have reported local control for small- and medium-sized choroidal melanomas treated with plaque radiotherapy in over 90% of cases [4, 5, 22–24]. Despite this success, 30–40% of patients experience significant radiation-related complications [4, 11]. The severity, location, and incidence of these complications are related to the type of radiation used, its method of delivery, total radiation dose, fractionation scale, and size and location of the tumor [11].

With the goal of minimizing treatment-related complications, investigators have studied methods to reduce the amount of radiation delivered to normal ocular tissues without compromise of local tumor control [11]. The use of lower energy radioisotopes (^125^I and ^106^Ru) and gold-shielded radiation-blocking devices are two measures which have reduced the radiation exposure to surrounding ocular structures [11, 16, 25]. Investigators have also studied the use of adjuvant hyperthermia as a means of reducing the amount of radiation necessary for tumor control [26–30]. In one study, with a mean followup interval of 22.2 months using plaque thermoradiotherapy, the minimum tumor radiation apex dose was reduced to 50 Gy, with a 97.7% local control rate [27]. Further investigation with longer followup is necessary to determine if plaque thermoradiotherapy reduces the rate of radiation-induced complications compared with plaque radiotherapy alone.

Based upon early studies, the minimum apical tumor treatment dose for plaque radiotherapy has been near 85 Gy. In addition, the COMS treated all tumors less than 5.0 mm to a minimum apex dose of 5.0 mm. Prior studies have not investigated the use of lower radiation doses for tumors with an apical height of less than 5.0 mm. The current study demonstrates that treating to the actual tumor height in choroidal melanomas less than 5.0 mm in apical height does not compromise local tumor control. 

Only one patient from treatment group 1 experienced tumor recurrence requiring enucleation after plaque radiotherapy. This difference was not significant. The incidence of local failure rates from various institutions has been reported at 15–20%; however, we have noted tumor recurrence in approximately 1% of cases after plaque radiotherapy [4, 5, 8, 22, 23]. Intraoperative plaque localization is performed in all surgical cases from this institution and may contribute to lower tumor recurrence rates [19, 21].

Only eight patients in the current study suffered metastatic disease (four from each treatment group). Of note, no patient with a tumor less than 2.5 mm in apical height developed metastatic disease. Previous studies have reported metastatic disease rates of 5.5–15.6% after ^125^I plaque radiotherapy with followup ranging from 46 to 64 months [4, 5, 8, 22, 23]. Studies with longer followup, such as this study, are therefore necessary to evaluate accurately the metastatic risk of using lower treatment doses. 

The mean followup interval in this study was 149.7 months, and while we did not show differences in visual acuity between the two treatment groups, the overall incidence of treatment-related complications was significantly higher in the group receiving higher radiation doses. It should be noted that the lack of a significant difference in visual acuity is likely to be related to the follow-up management for all patients that presented with macular edema secondary to radiation retinopathy. This management included anti-VEGF and intravitreal steroidal therapy. Only radiation retinopathy and visually significant cataract formation were found to be significantly different when complications were analyzed separately.

As demonstrated in the current study, lower radiation treatment doses may be equally effective in local tumor control and prevention of metastasis for small-medium choroidal melanomas when compared with standard treatment doses. Furthermore, use of less radiation may lower the incidence of radiation-related sight-threatening complications. We have found no difference in local tumor control, metastasis, and visual outcomes when treating to the true apical height of tumors smaller than 5.0 mm. Furthermore, treatment-related complications were significantly lower in patients receiving lower doses of radiation. Limitations of the current study should be recognized including the small sample, followup intervals, and single institutional review. 

## Figures and Tables

**Table 1:** Tumor characteristics.

Tumor characteristic	Treatment group			*P* value
	Group 1 (Apical height)	Group 2 (5 mm)	Total	(Pearson's chi-squared test)
Largest diameter of tumor (mm)				
Mean	10.7	10.8	10.7	0.9
Standard deviation	2.8	3.1	2.9	
Ultrasound height (mm)				
Mean	3.3	3.2	3.3	0.9
Standard deviation	0.76	0.82	0.78	
Location				
Posterior to equator	27	16	43	
Anterior to equator	15	13	28	0.4
Both anterior and posterior	6	8	14	
Mushroom shaped				
Yes	12	4	16	0.4
No	39	32	71	

**Table 2:** Tumor recurrence, metastasis, mortality, enucleations, and followup interval.

	Treatment group			*P* value
	Group 1 (Apical height)	Group 2 (5 mm)	Total	(Pearson's chi-squared test)
Tumor recurrence	1	0	1	0.40
Metastasis	4	4	8	0.99
Mortality*	4	4	8	0.99
Enucleations	2	1	3	0.56
Followup				
Mean	145.2	151.1	149.7	0.90

*All of these deaths were related to uveal melanoma.

**Table 3:** Visual acuity outcomes.

	Visual acuity (number (%))			*P* value
	≤20/40	20/40–20/200	≤20/200	(Pearson's chi-squared test)
Pretreatment				
Group 1	43 (78)	8 (15)	4 (7)	0.19
Group 2	25 (63)	8 (20)	7 (18)	
6 month				
Group 1	38 (69)	9 (16)	8 (15)	0.50
Group 2	24 (60)	7 (18)	9 (22)	
12 month				
Group 1	39 (72)	6 (11)	9 (17)	0.7
Group 2	27 (68)	3 (8)	10 (25)	
24 month				
Group 1	28 (52)	9 (17)	17 (31)	0.5
Group 2	26 (65)	2 (5)	12 (30)	

*One eye enucleated at 10 months in group 1.

**Table 4:** Complications from iodine-125 plaque irradiation at 10 years.

Complication	Number of cases (%)	Mean time interval from treatment (months ± standard dev.)	*P* values (Pearson's chi-squared test)
Radiation retinopathy	45	27 ± 15	
Group 1	14 (20)	29 ± 18	<0.001
Group 2	31 (78)	25 ± 10	
Radiation papillopathy	23	29 ± 16	
Group 1	11 (20)	29 ± 20	0.261
Group 2	12 (30)	29 ± 16	
Cataract	13	29 ± 22	
Group 1	4 (7)	33 ± 22	0.04
Group 2	9 (23)	27 ± 23	
Strabismus	14	5 ± 7	
Group 1	6 (11)	2 ± 2	0.54
Group 2	8 (20)	7 ± 9	
Vitreous hemorrhage	7	23 ± 20	
Group 1	2 (4)	20 ± 16	0.19
Group 2	5 (12)	25 ± 23	
Exudative RD	3	38 ± 18	
Group 1	1 (2)	59	0.18
Group 2	2 (5)	28 ± 1	
Overall treatment-related complications*	33		
Group 1	14 (27)		0.00053
Group 2	39 (98)		

*Patients experiencing one or more of the following treatment-related complications: radiation retinopathy, radiation papillopathy, visually significant cataract, or vitreous hemorrhage.

**There were no cases of neovascular glaucoma.
